# Preoperative low Geriatric Nutritional Risk Index increases intensive care unit admission risk in patients undergoing gastrointestinal tumor surgery

**DOI:** 10.3389/fnut.2026.1731167

**Published:** 2026-05-28

**Authors:** Wei-De Lin, Bi-Xia Lin, Jun-Fan Chen, Xiao-Ping Huang

**Affiliations:** 1 Department of Anesthesiology, The First Hospital of Putian City, Putian, China; 2 Department of Ultrasonography, The First Hospital of Putian City, Putian, China; 3 Department of Medical Equipment, The First Hospital of Putian City, Putian, China

**Keywords:** gastrointestinal tumor, Geriatric Nutritional Risk Index, intensive care unit, intensive care unit admission risk, retrospective study

## Abstract

**Background:**

Gastrointestinal tumors are highly prevalent malignant tumors worldwide, and surgery is their primary treatment modality. Although the Geriatric Nutritional Risk Index (GNRI), a simple nutritional assessment tool, has been associated with prognosis in various diseases, its impact on ICU admission risk following gastrointestinal tumor surgery remains unclear. This study aims to investigate the relationship between preoperative GNRI and postoperative ICU admission risk.

**Methods:**

This retrospective cohort study analyzed 9,045 gastrointestinal tumor surgery patients from the INSPIRE database. Multivariable logistic regression with stepwise confounder adjustment was used to examine the GNRI-ICU admission association. Generalized additive models explored nonlinear relationships, while subgroup and sensitivity analyses verified result robustness.

**Results:**

Multivariable regression analysis demonstrated that preoperative GNRI decrease was significantly associated with increased risk of ICU admission in patients after gastrointestinal tumor surgery. The severe nutritional risk group (GNRI < 82) had a 2.01-fold higher risk of ICU admission compared to the no-risk group (GNRI > 98) (95% CI: 1.09–3.69). Nonlinear analysis identified GNRI = 89 as a key threshold (*p* < 0.001), beyond which each unit increase in GNRI reduced the risk by 12.6%. Subgroup analysis and sensitivity analyses both confirmed the robustness of the results.

**Conclusion:**

Preoperative GNRI is an independent predictor for ICU admission following gastrointestinal tumor surgery, demonstrating a significant non-linear relationship with a critical threshold at GNRI = 89. The categorical risk stratification (GNRI < 82) provides clinically meaningful information for perioperative risk assessment.

## 1 Introduction

Gastrointestinal tumors are a heterogeneous group of neoplasms originating from the epithelial or mesenchymal tissues of the digestive tract, encompassing multiple anatomical sites from the esophagus to the rectum. Their incidence and mortality rates remain high worldwide (1, 2). Surgical resection is the primary treatment for these malignancies. With advancements in surgical techniques and optimization of perioperative management, patient survival rates have significantly improved (3). The application of new technologies such as minimally invasive surgery, prehabilitation, and precision medicine has further brought revolutionary changes to gastrointestinal tumor surgery (4, 5). Studies have shown that preoperative nutritional intervention helps improve patients’ functional reserve and reduce postoperative complications (5, 6). Nevertheless, postoperative complications and functional impairment remain core issues leading to adverse clinical outcomes, prolonged hospital stays, and increased healthcare resource consumption. Among these, clinical deterioration requiring transfer to the Intensive Care Unit (ICU) postoperatively is a key event for assessing major perioperative risks. It not only directly impacts patient prognosis but also places a heavy burden on the healthcare system. Therefore, accurately identifying high-risk patients prone to ICU admission and implementing targeted interventions are of great significance for optimizing clinical decision-making and healthcare resource allocation.

The Geriatric Nutritional Risk Index (GNRI) is a simple nutritional screening tool based on serum albumin and body weight, widely used to assess the nutritional status and related risks in elderly patients (7, 8). Studies have shown that the GNRI value is negatively correlated with nutritional risk, which is directly related to decreased serum albumin levels and weight loss (8, 9). With the intensification of global population aging, malnutrition has become a significant health issue for the elderly. As a key modifiable factor affecting prognosis, it is closely related to the outcomes of various diseases (10, 11). By quantifying nutritional risk, GNRI provides an effective predictive tool for clinical practice, and its application value in various fields such as cardiovascular diseases, tumors, and orthopedic diseases is increasingly attracting attention (12–15). Numerous studies have shown that GNRI can not only identify the risk of malnutrition but also predict postoperative complications, disease prognosis, and quality of life, holding significant importance for improving clinical outcomes in elderly patients (16, 17). Although existing research has preliminarily established the correlation between GNRI and the prognosis of various diseases, its specific impact on the risk of ICU admission in postoperative patients with gastrointestinal tumors lacks in-depth exploration. Therefore, based on real-world data, this study aims to focus on analyzing the impact of preoperative GNRI levels on the risk of ICU admission in postoperative patients with gastrointestinal tumors, in order to provide a reference for clinical risk stratification and the development of intervention strategies.

## 2 Materials and methods

### 2.1 Study population

This study utilized the INSPIRE database. This database contains data from approximately 130,000 patients who underwent surgical anesthesia at Seoul National University Hospital between 2011 and 2020 (18). We extracted 9,045 patients who underwent gastrointestinal tumor surgery as the study cohort. This study adhered to the ethical principles of the Declaration of Helsinki. All data were sourced from the publicly available and fully de-identified INSPIRE database, containing no personal identifiable information, and researchers were unable to identify individual participants. The study protocol was reviewed and approved by the Institutional Review Board of Seoul National University Hospital (IRB No. H-2210-078-1368). The IRB determined that, given the retrospective use of fully anonymized data from a public database, the study met the criteria for exemption from ongoing ethical review and waived the requirement for written informed consent. The study report was prepared following the STROBE guidelines for observational studies (19).

### 2.2 Covariates and outcome

We used SQL for data extraction. The included covariates were age, gender, american society of anesthesiologists classification (ASA classification), emergency operation (EmOP), general anesthesia (GA), anesthesia time (AT), heart rate (HR), systolic blood pressure (SBP), diastolic blood pressure (DBP), respiratory rate (Resp), pulse oximetry derived oxygen saturation (SpO2), temperature (T), body mass index (BMI), hemoglobin (Hb), platelet count (PLT), white blood cell count (WBC), glucose, albumin (Alb), serum creatinine (Scr), total bilirubin (TBIL), sodium (Na), potassium (K), calcium (Ca), chlorine (Cl), vasoactive agents (VAAs), hypertension (HTN), diabetes mellitus (DM), and cardiovascular disease (CVD). GNRI was calculated as 1.489 × albumin (measured in g/L) + 41.7 × (actual body weight/ideal body weight), with ideal body weight determined by the Lorentz equations: for men, height (cm) - 100 - [(height (cm) - 150)/4]; for women, height (cm) - 100 - [(height (cm) - 150)/2.5]. When actual weight exceeded ideal weight, the ratio was set to 1. Based on established nutritional risk thresholds in elderly populations, GNRI values were categorized into four risk levels: major risk (GNRI < 82), moderate risk (82 ≤ GNRI < 92), low risk (92 ≤ GNRI ≤ 98), and no risk (GNRI > 98) (20). The study used postoperative ICU admission as the outcome measure. Postoperative ICU admission was defined as the first transfer to the intensive care unit at any time point from the end of surgery until hospital discharge.

### 2.3 Statistical analysis

Continuous variables are presented as mean ± standard deviation or median (interquartile range), while categorical variables are presented as number (percentage). Ten extreme outliers in BMI (values ranging from 142 to 6,500) were identified as physiologically implausible and were excluded from all analyses, as they likely represented data entry errors. Inter-group comparisons were performed using the t-test, Mann–Whitney U test, Chi-square test, or Fisher’s exact test, as appropriate. All missing data were handled using complete case analysis (missing data ranged from 0.1 to 4.8%). To ensure model stability, we performed multicollinearity diagnostics on all covariates before modeling, calculating the Variance Inflation Factor (VIF). All VIFs were confirmed to be below 5 (Supplementary Table S1), and a pairwise correlation matrix showed no strong correlations among the variables (Supplementary Figure S1), indicating that collinearity was within an acceptable range.

To assess the association between GNRI and ICU admission risk, we first performed univariable logistic regression analysis. Subsequently, a series of multivariable models were constructed to progressively adjust for confounding factors: Model I adjusted for age and sex; Model II, building on Model I, further included covariates with a *p*-value < 0.1 in the univariable analysis or those that altered the effect size by >10%; Model III was the fully adjusted model.

We applied a generalized additive model to explore the relationship between GNRI and the risk of ICU admission in patients after gastrointestinal tumor surgery (21). To determine whether the relationship between GNRI levels and ICU admission risk was consistent across subgroups of patients after gastrointestinal tumor surgery, we conducted interaction and subgroup analyses based on various baseline characteristics such as age, sex, ASA classification, use of vasoactive agents, hypertension, diabetes, and cardiovascular disease. Propensity score matching (PSM) was used to control for potential confounding bias. A logistic regression model was employed to calculate propensity scores, incorporating all baseline covariates. A 1:1 nearest-neighbor matching algorithm was applied, using a caliper width of 0.2 times the standard deviation of the logit-transformed propensity score. This process successfully matched 697 pairs (1,394 patients) out of the original 9,045 individuals. Standardized mean differences (SMDs) were used to assess balance before and after matching, with an SMD of less than 0.1 considered an acceptable threshold for adequate balance. After matching, all covariates achieved SMD < 0.1, indicating good balance between the two groups. Additionally, to assess the potential bias introduced by missing data, we performed multiple imputation (with five imputed datasets) as a sensitivity analysis. Separately, to evaluate the impact of the ten extreme BMI outliers, we excluded these outliers and re-ran the primary logistic regression models.

All analyses were performed using R and Free Statistics software, with a two-sided *p*-value < 0.05 defined as statistically significant (22).

## 3 Results

### 3.1 Study subjects and demographic characteristics

**Figure 1:**
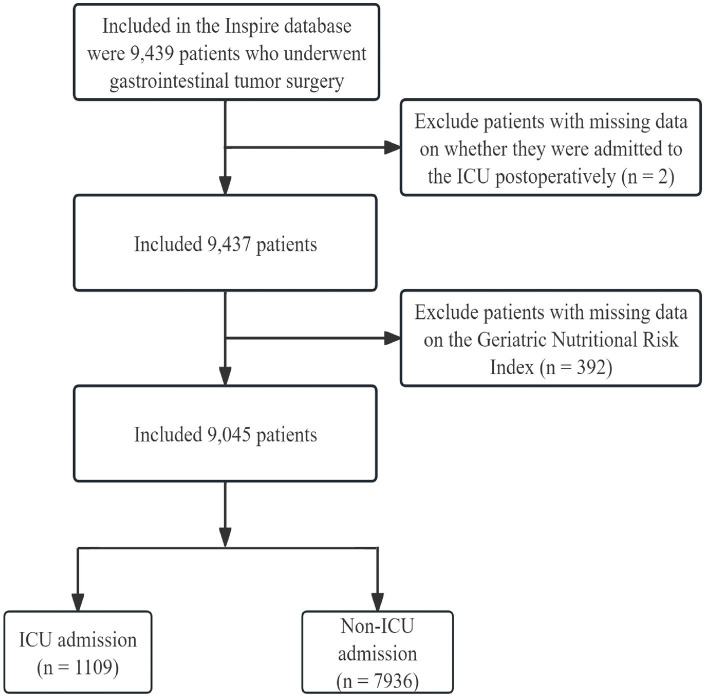
Schematic representation of the participant selection process and distribution of participant groups.

This study initially included 9,439 patients who underwent gastrointestinal tumor surgery (age range 18–90 years). For patients who underwent multiple surgeries, only data from the first surgery were included. After excluding 2 patients with missing ICU admission data and 394 patients with missing GNRI data, the final study cohort comprised 9,045 patients who underwent gastrointestinal tumor surgery at Seoul National University Hospital (Figure 1).

Table 1 summarizes the basic clinical data and demographic characteristics of the included subjects. The risk of ICU admission in this cohort was 12.3%. The mean age of the patients was 62.5 ± 11.7 years, with 63.2% being male. Emergency operations accounted for 6.6%, and general anesthesia was used in 97% of the cases. As shown in Supplementary Figure S2, the distribution of baseline characteristics across GNRI groups was visualized using bar plots: Panels A and B: Distribution differences of continuous variables across GNRI groups; Panel C: Overall distribution characteristics of categorical variables in the entire cohort. Further intergroup comparisons revealed that compared to the low GNRI group, the high GNRI group had shorter anesthesia time, lower heart rate, higher levels of hemoglobin, body mass index, platelet count, and albumin, while demonstrating lower white blood cell count and blood glucose levels. All data were sourced from the INSPIRE database.

**Table 1:** Baseline characteristics of participants.

Variables	Total (*n* = 9,045)	GNRI< 82 (*n* = 912)	82 ≤ GNRI< 92 (*n* = 2,454)	92 ≤ GNR≤ 98 (*n* = 2065)	GNRI> 98 (*n* = 3,614)	*p* value
Age (years)	62.5 ± 11.7	65.8 ± 12.5	63.3 ± 11.9	62.1 ± 11.7	61.3 ± 11.2	< 0.001
Gender, *n* (%)						< 0.001
Female	3,328 (36.8)	341 (37.4)	981 (40)	788 (38.2)	1,218 (33.7)	
Male	5,717 (63.2)	571 (62.6)	1,473 (60)	1,277 (61.8)	2,396 (66.3)	
ASA classification, *n* (%)						< 0.001
I	2,682 (30.0)	218 (24.6)	769 (31.8)	640 (31.4)	1,055 (29.4)	
II	5,600 (62.7)	547 (61.8)	1,462 (60.5)	1,272 (62.3)	2,319 (64.7)	
≥III	645 (7.2)	120 (13.6)	186 (7.7)	129 (6.3)	210 (5.9)	
EmOP, *n* (%)						< 0.001
No	8,446 (93.4)	787 (86.3)	2,269 (92.5)	1942 (94)	3,448 (95.4)	
Yes	599 (6.6)	125 (13.7)	185 (7.5)	123 (6)	166 (4.6)	
GA, *n* (%)						< 0.001
No	268 (3.0)	16 (1.8)	32 (1.3)	54 (2.6)	166 (4.6)	
Yes	8,777 (97.0)	896 (98.2)	2,422 (98.7)	2011 (97.4)	3,448 (95.4)	
AT (min)	200.0 (130.0, 260.0)	210.0 (145.0, 275.0)	215.0 (155.0, 270.0)	200.0 (135.0, 255.0)	180.0 (115.0, 255.0)	< 0.001
HR (bpm)	76.2 ± 13.8	81.2 ± 15.7	76.5 ± 13.9	75.1 ± 13.4	75.3 ± 13.1	< 0.001
SBP (mmHg)	126.3 ± 17.3	123.4 ± 19.2	125.0 ± 17.8	126.0 ± 17.0	128.0 ± 16.3	< 0.001
DBP (mmHg)	75.9 ± 10.8	73.6 ± 11.4	74.5 ± 11.1	75.5 ± 10.6	77.6 ± 10.4	< 0.001
Resp (bpm)	17.8 ± 1.2	17.9 ± 1.6	17.7 ± 1.2	17.7 ± 1.0	17.8 ± 1.1	< 0.001
SpO_2_ (%)	97.4 ± 1.9	97.5 ± 1.9	97.4 ± 1.9	97.4 ± 1.9	97.4 ± 1.9	0.243
T (°C)	36.4 ± 0.4	36.5 ± 0.5	36.4 ± 0.4	36.3 ± 0.4	36.3 ± 0.4	< 0.001
BMI (kg/m^2^)	23.0 ± 3.7	19.3 ± 4.1	22.2 ± 3.5	22.9 ± 3.4	24.4 ± 3.0	< 0.001
Hb (g/dL)	12.3 ± 1.9	10.5 ± 1.8	11.6 ± 1.7	12.4 ± 1.7	13.1 ± 1.6	< 0.001
PLT(×10^9^/L)	231.6 ± 76.1	245.1 ± 97.4	228.2 ± 82.3	226.7 ± 72.4	233.3 ± 66.6	< 0.001
WBC (×10^9^/L)	8.2 ± 3.5	9.4 ± 4.0	9.3 ± 3.7	8.4 ± 3.5	7.1 ± 2.7	< 0.001
Glucose (mg/dL)	118.0 ± 38.4	121.6 ± 44.4	118.6 ± 38.2	116.8 ± 36.6	117.5 ± 37.8	0.013
Alb(g/dL)	3.7 ± 0.5	2.8 ± 0.3	3.3 ± 0.3	3.7 ± 0.2	4.1 ± 0.2	< 0.001
Scr (mg/dL)	0.8 (0.7, 1.0)	0.7 (0.6, 0.9)	0.8 (0.7, 0.9)	0.8 (0.7, 0.9)	0.8 (0.7, 1.0)	< 0.001
TBIL (μmol/L)	0.7 (0.5, 1.0)	0.5 (0.5, 0.8)	0.7 (0.5, 1.0)	0.7 (0.5, 1.0)	0.5 (0.5, 0.8)	< 0.001
Na (mmol/L)	139.3 ± 2.7	137.5 ± 3.7	139.2 ± 2.9	139.5 ± 2.5	139.8 ± 2.3	< 0.001
K (mmol/L)	4.0 (3.8, 4.4)	4.0 (3.8, 4.4)	4.0 (3.8, 4.4)	4.0 (3.8, 4.2)	4.2 (3.8, 4.4)	< 0.001
Ca (mmol/L)	8.6 ± 0.6	8.0 ± 0.5	8.3 ± 0.4	8.6 ± 0.4	9.0 ± 0.4	< 0.001
Cl (mmol/L)	104.1 ± 3.4	103.5 ± 4.3	104.2 ± 3.6	104.4 ± 3.3	103.9 ± 3.0	< 0.001
VAAs, *n* (%)						< 0.001
No	8,818 (97.5)	888 (97.4)	2,411 (98.2)	2026 (98.1)	3,493 (96.7)	
Yes	227 (2.5)	24 (2.6)	43 (1.8)	39 (1.9)	121 (3.3)	
HTN, *n* (%)						0.124
No	8,139 (90.0)	828 (90.8)	2,226 (90.7)	1866 (90.4)	3,219 (89.1)	
Yes	906 (10.0)	84 (9.2)	228 (9.3)	199 (9.6)	395 (10.9)	
DM, *n* (%)						0.095
No	8,125 (89.8)	829 (90.9)	2,202 (89.7)	1877 (90.9)	3,217 (89)	
Yes	920 (10.2)	83 (9.1)	252 (10.3)	188 (9.1)	397 (11)	
CVD, *n* (%)						0.971
No	8,697 (96.2)	877 (96.2)	2,362 (96.3)	1982 (96)	3,476 (96.2)	
Yes	348 (3.8)	35 (3.8)	92 (3.7)	83 (4)	138 (3.8)	

Continuous variables are presented as mean ± SD or median (quartile), while categorical variables are presented as absolute numbers (percentages).

ASA classification, American Society of Anesthesiologists classification; EmOP, Emergency Operation; GA, General anesthesia; AT, Anesthesia Time; HR, Heart rate; SBP, systolic blood pressure; DBP, diastolic blood pressure; Resp., respiratory; SpO_2_, pulse oximetry derived oxygen saturation; T, Temperature; BMI, body mass index; Hb, hemoglobin; PLT, Platelet Count; WBC, white blood cell; Alb, albumin; Scr, serum creatinine; TBIL, total bilirubin; Na, Sodium; K, Potassium; Ca, Calcium; Cl, Chlorine; VAAs, Vasoactive Agents; HTN, Hypertension; DM, diabetes mellitus; CVD, Cardiovascular Disease.

### 3.2 Logistic regression analysis of postoperative ICU admission risk

Univariable regression analysis identified factors associated with ICU admission risk (Supplementary Table S2). The analysis showed that Age, Gender, ASA classification, EmOP, GA, AT, DBP, SpO2, BMI, Hb, PLT, Glucose, Alb, Scr, Na, K, Cl, VAAs, HTN, DM, and CVD were all significant predictors of ICU admission risk (*p* < 0.05).

We further analyzed the association between GNRI and postoperative ICU admission risk in patients with gastrointestinal tumors using logistic regression models (Table 2). In this study, GNRI was reciprocally transformed to make the interpretation of the odds ratio more intuitive. In the unadjusted crude model, when GNRI was treated as a continuous variable, each unit decrease in its value was associated with a corresponding increase in the risk of postoperative ICU admission (Crude model, OR = 1.01; 95% CI: 1.01–1.02; *p* < 0.001). This positive correlation persisted in the partially adjusted model and remained significant in the fully adjusted Model 3 (Model 3, OR = 1.02; 95% CI: 1.01–1.03; *p* < 0.001).

**Table 2:** The relationship between GNRI and ICU admission in different models.

Variable	Crude model		Model I		Model II		Model III	
	OR (95% CI)	*p-*value	OR (95% CI)	*p-*value	OR (95% CI)	*p-*value	OR (95% CI)	*p-*value
GNRI (continuous)	1.01 (1.01 ~ 1.02)	<0.001	1.01 (1.01 ~ 1.02)	<0.001	1.02 (1.01 ~ 1.03)	<0.001	1.02 (1.01 ~ 1.03)	<0.001
GNRI quartiles								
GNRI >98	Ref		Ref		Ref		Ref	
92 ≤ GNR ≤ 98	0.98 (0.82 ~ 1.16)	0.798	0.94 (0.79 ~ 1.13)	0.519	1.12 (0.85 ~ 1.47)	0.411	1.12 (0.86 ~ 1.47)	0.398
82 ≤ GNRI <92	1.04 (0.88 ~ 1.22)	0.643	0.94 (0.79 ~ 1.11)	0.457	0.98 (0.66 ~ 1.44)	0.901	0.98 (0.66 ~ 1.45)	0.921
GNRI <82	2.32 (1.92 ~ 2.79)	<0.001	1.89 (1.55 ~ 2.3)	<0.001	1.99 (1.08 ~ 3.66)	0.027	2.01 (1.09 ~ 3.69)	0.025
P for trend		<0.001		<0.001		0.471		0.447

Crude model was not adjusted.

Model 1 was adjusted for age + Gender.

Model 2 was adjusted for model 1 + ASA classification + EmOP + GA + AT + HR + PLT + DBP + SpO_2_ + Hb + BMI + Glucose + Alb + Scr + TBIL + Na + K + Ca + Cl + VAAs + HTN + DM + CVD.

Model 3 was adjusted for model 2 + SBP + Resp + T + WBC.

ASA classification, American Society of Anesthesiologists classification; EmOP, Emergency Operation; GA, General anesthesia; AT, Anesthesia Time; HR, Heart rate; SBP, systolic blood pressure; DBP, diastolic blood pressure; Resp., respiratory; SpO_2_, pulse oximetry derived oxygen saturation; T, Temperature; BMI, body mass index; Hb, hemoglobin; PLT, Platelet Count; WBC, white blood cell; Alb, albumin; Scr, serum creatinine; TBIL, total bilirubin; Na, Sodium; K, Potassium; Ca, Calcium; Cl, Chlorine; VAAs, Vasoactive Agents; HTN, Hypertension; DM, diabetes mellitus; CVD, Cardiovascular Disease.

**Figure 2:**
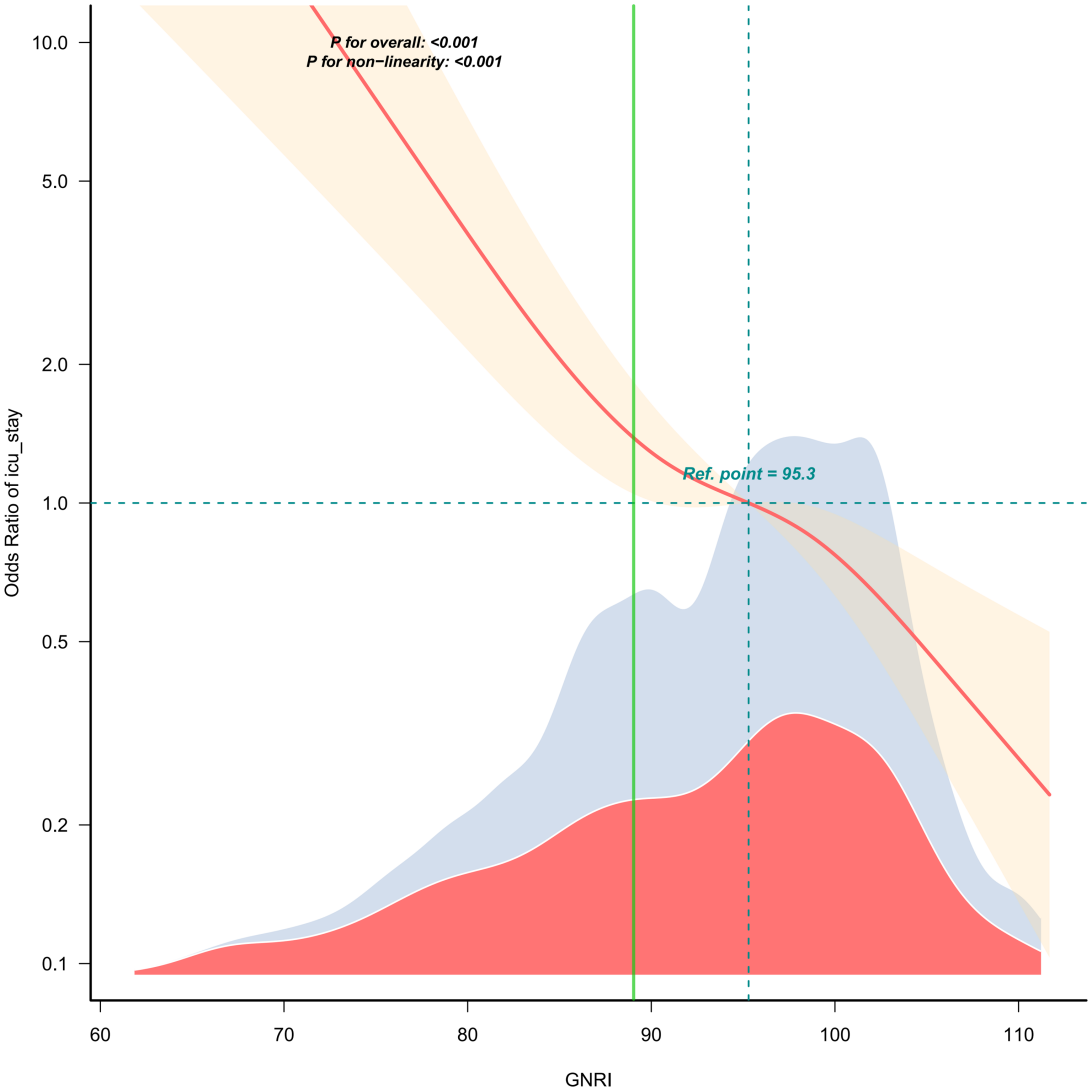
Restricted cubic spline of the association of GNRI with ICU admission risk following surgery for gastrointestinal tumors. The vertical solid green line at GNRI = 89 indicates the key threshold identified by the threshold analysis. The nonlinear dose–response relationship between GNRI and the risk of ICU admission in postoperative patients with gastrointestinal tumors. Adjustments were made for age, gender, ASA classification, EmOP, GA, AT, HR, PLT, DBP, SpO_2_, Hb, BMI, Glucose, Alb, Scr, TBIL, Na, K, Ca, Cl, VAAs, HTN, DM, CVD, SBP, Resp, T, WBC. The solid red line indicates the estimated risk of ICU admission, while the light yellow shaded area represents the 95% confidence interval. The distribution of GNRI for patients who were admitted to the ICU is represented by the red shaded area. The distribution for patients who were not admitted to the ICU is represented by the gray shaded area. Data for 99% of the study participants are displayed.

When GNRI was treated as a categorical variable, in the unadjusted model, patients in the GNRI < 82 group had a 2.32 times higher risk of postoperative ICU admission compared to the GNRI > 98 group (Crude model, OR = 2.32; 95% CI: 1.92–2.79; p < 0.001). This association remained significant after partial adjustment for variables. In the fully adjusted Model 3, the risk for the GNRI < 82 group was still 2.01 times that of the GNRI > 98 group (Model 3, OR = 2.01; 95% CI: 1.09–3.69; *p* = 0.025). Furthermore, restricted cubic spline analysis revealed a non-linear dose–response relationship between GNRI levels and the risk of ICU admission in patients after gastrointestinal tumor surgery (*p* < 0.001) (Figure 2). Threshold analysis indicated that when GNRI ≥ 89, the risk of ICU admission in patients after gastrointestinal tumor surgery significantly decreased (OR = 0.874; 95% CI: 0.825–0.927; *p* < 0.001). That is, above this threshold, for each unit increase in GNRI, the patient’s risk of ICU admission decreased by 12.6%. Of note, although the per-unit decrease in GNRI was associated with a statistically significant 2% increase in ICU admission risk (OR = 1.02), this effect size is clinically modest. Therefore, the categorical risk stratification (GNRI < 82) and the threshold effect (GNRI = 89) are more relevant for clinical decision-making (Table 3).

**Table 3:** Threshold effect of Geriatric Nutritional Risk Index on ICU admission risk.

In-hospital mortality			
Turning point	OR	95%CI	*p*-value
GNRI <89	0.983	0.945,1.022	0.3892
GNRI ≥89	0.874	0.825,0.927	< 0.001
Likelihood Ratio test			0.022

OR, odds ratio; CI, confidence interval. Adjustment factors included age, Gender, ASA classification, EmOP, GA, AT, HR, PLT, DBP, SpO_2_, Hb, BMI, Glucose, Alb, Scr, TBIL, Na, K, Ca, Cl, VAAs, HTN, DM, CVD, SBP, Resp, T, WBC.

### 3.3 Subgroup analysis

**Figure 3:**
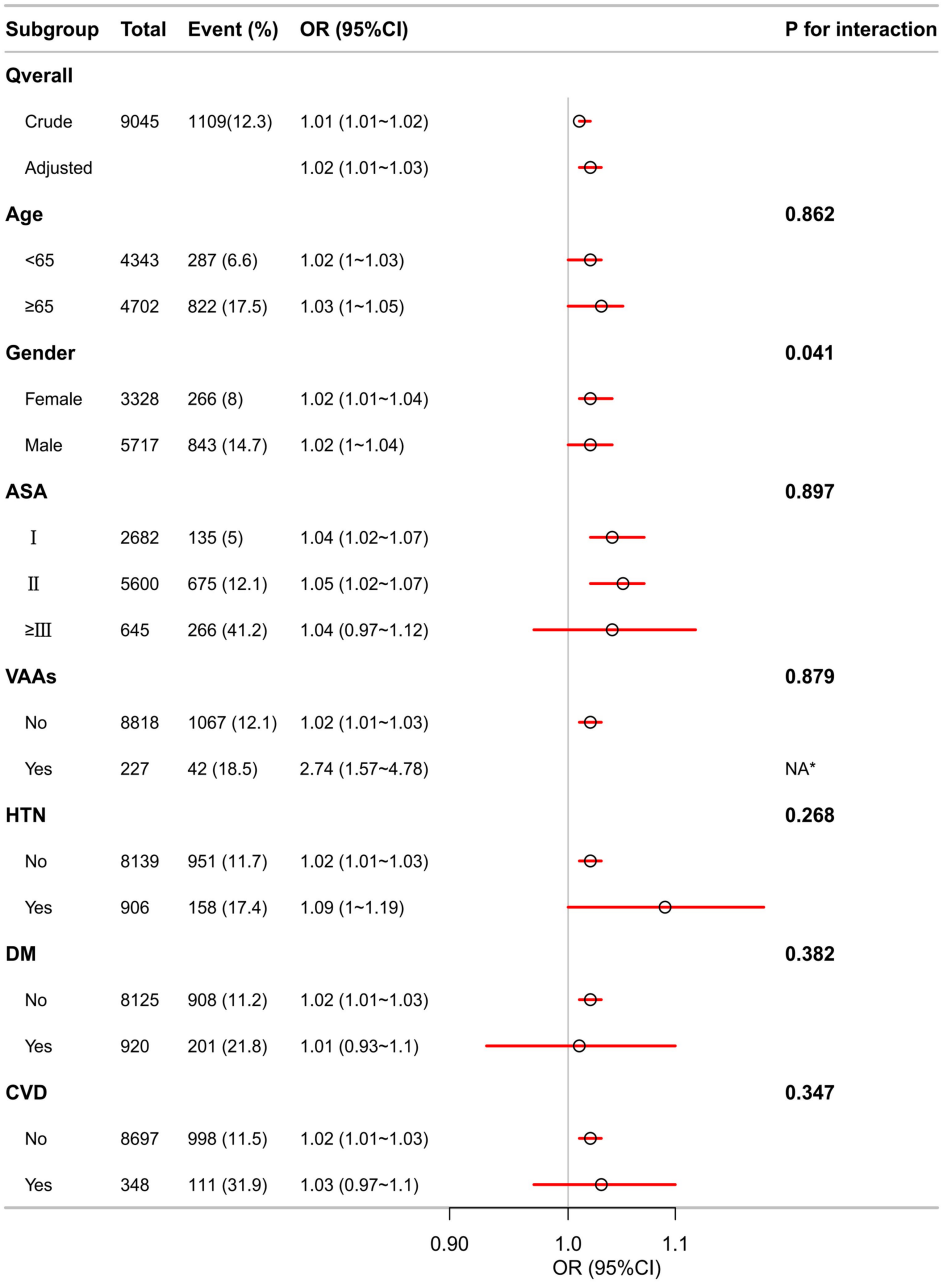
Subgroup analysis of GNRI and postoperative ICU admission risk in gastrointestinal tumor patients. The analysis was adjusted for age, gender, ASA classification, EmOP, GA, AT, HR, PLT, DBP, SpO_2_, Hb, BMI, Glucose, Alb, Scr, TBIL, Na, K, Ca, Cl, VAAs, HTN, DM, CVD, SBP, Resp, T, WBC.

This study validated the consistency of the association between GNRI and postoperative ICU admission risk across different subgroups through stratification and interaction analyses. The analysis was conducted with stratification and interaction effect analysis based on age (<65/≥65 years), sex (Female/Male), ASA classification (I/II/≥III), VAAs (No/Yes), hypertension (No/Yes), diabetes (No/Yes), and cardiovascular disease (No/Yes). An interaction effect was found for sex on postoperative ICU admission risk (*p* = 0.041), while no statistically significant interaction effects were found for the other subgroups (Figure 3).

### 3.4 Sensitivity analysis

**Figure 4:**
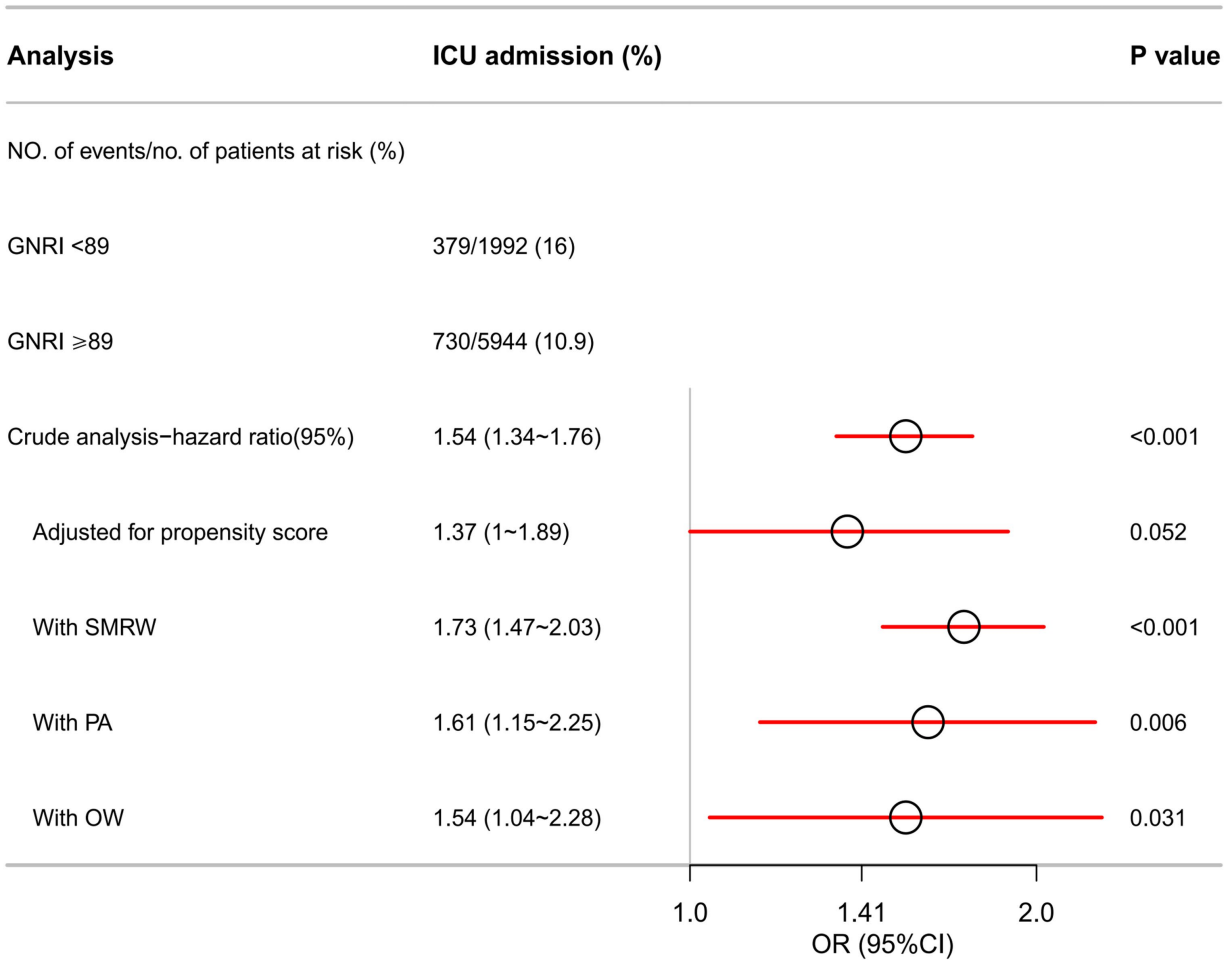
Association between GNRI and ICU admission risk presented as odds ratios across different propensity score adjustment methods.

To reduce potential confounding factors, we divided patients into two groups based on the threshold identified by the threshold analysis: GNRI ≥ 89 and GNRI < 89. Distribution plots of standardized mean differences for each variable before and after propensity score adjustment were generated (Supplementary Figure S3). The results showed that after applying various adjustment methods, including PSM, Standardized Mortality Ratio Weighting (SMRW), Pairwise Algorithmic (PA) weighting, and Overlap Weight (OW), the differences between groups for the vast majority of variables were significantly reduced, indicating effective control of confounding factors. Univariable analysis, SMRW, PA, and OW regression analyses consistently indicated that GNRI < 89 was significantly associated with an increased risk of ICU admission (OR range = 1.37–1.73, all *p* < 0.05). In the PSM analysis, the association did not reach statistical significance (OR = 1.37, 95% CI: 1.00–1.89, *p* = 0.052). The loss of statistical power due to sample size reduction after matching may have contributed to this borderline result. Nevertheless, other sensitivity analyses (SMRW, PA, OW, multiple imputation, and BMI outlier exclusion) consistently showed significant associations, supporting the robustness of our findings (Figure 4).

In the sensitivity analysis, we performed multiple imputation to assess potential bias introduced by missing data. The results were consistent with the complete case analysis: when GNRI was treated as a continuous variable, the association with ICU admission remained statistically significant in the fully adjusted model (OR = 1.02, 95% CI: 1.01–1.03, *p* < 0.001); when GNRI was treated as a categorical variable, patients in the GNRI < 82 group had a significantly higher risk of ICU admission compared with the GNRI > 98 group (fully adjusted model, OR = 1.82, 95% CI: 1.01–3.29, *p* = 0.048) (Supplementary Table S3). This indicates that the association between GNRI and ICU admission remained statistically significant.

We further conducted a sensitivity analysis excluding ten extreme BMI outliers. After excluding these outliers, the association between GNRI and ICU admission remained statistically significant, and the direction of the effect was consistent with the primary analysis (Supplementary Table S4).

## 4 Discussion

This study confirms that the GNRI is an independent predictor of ICU admission risk following gastrointestinal tumor surgery. The analysis results show a negative correlation between GNRI and ICU admission risk, which remained significant after multiple adjustments and sensitivity analyses. Dose–response relationship analysis identified GNRI = 89 as a key critical value, with patient risk significantly increasing below this threshold. Subgroup analysis results indicated that this association demonstrated good consistency across most pre-specified subgroups. These findings suggest that incorporating nutritional risk assessment into the perioperative management process can aid in the earlier identification of patients at higher risk for ICU admission. This provides important evidence-based support for developing prehabilitation strategies targeted at high-risk patients.

Previous studies have confirmed that a low GNRI is associated with increased complication rates and mortality in various diseases (such as heart failure, diabetes, pancreatic cancer, etc.) and following various surgeries (such as orthopedic, cardiovascular, and gastrointestinal surgeries) (23–29). Okabayashi et al. (30) confirmed that GNRI has benefits in reducing complications and shortening hospital stays for patients undergoing hepatectomy; Burden et al. (31) further demonstrated that for colorectal cancer patients, oral nutritional supplementation significantly alleviated weight loss and reduced the risk of postoperative infections. Our research findings extend the existing evidence, indicating that GNRI is not only an effective prognostic indicator for survival but is also closely related to the risk of acute clinical deterioration events (such as ICU admission). This study systematically evaluated the association between GNRI and the risk of ICU admission in patients after gastrointestinal tumor surgery. Our results indicate that GNRI is a strong risk marker for this acute clinical event. The underlying biological mechanisms may involve multiple levels: Firstly, the malnourished state reflected by a low GNRI directly impairs immune function and weakens the patient’s tolerance to surgical trauma and postoperative stress (32). Secondly, low albumin levels are not only a marker of insufficient nutritional intake but also a manifestation of systemic inflammatory response (33, 34). In cancer patients, a persistent inflammatory state can accelerate protein catabolism, further worsening nutritional status, thereby counteracting the anti-cancer benefits of albumin itself, promoting tumor progression, and forming a self-sustaining vicious cycle (35). Furthermore, tumor-associated inflammation induces cachexia by upregulating cytokine levels; this pathological state not only reduces patient tolerance but can also lead to adverse clinical outcomes (25, 36). This dual hit of “nutrition-inflammation” weakens tissue repair capacity and immune function, making patients more susceptible to severe postoperative complications such as infection and anastomotic leakage, ultimately increasing the risk of clinical deterioration requiring transfer to the ICU for advanced life support.

It is noteworthy that the nutritional and inflammatory status reflected by GNRI also influences the long-term biological behavior of tumors (37). Previous studies have shown that a decreased GNRI suggests that the tumor may have higher invasiveness (38), indicating that this index can serve as a surrogate marker for assessing tumor progression and holds potential value in prognosis prediction. This viewpoint has been corroborated by multiple studies across different disease backgrounds, further supporting the broad application prospects of GNRI in tumor prognosis assessment. For example, a study from the United States found that a low GNRI is an independent predictor of 30-day complications following nephrectomy for renal tumors (39). Another Japanese cohort study found that in patients with metastatic colorectal cancer receiving systemic chemotherapy, the pre-treatment GNRI was an independent prognostic factor for overall survival. A lower GNRI value was significantly associated with poorer performance status, higher tumor burden, fewer opportunities for surgical intervention, and effectively predicted poor survival outcomes (40). Furthermore, a Chinese study on patients after hepatocellular carcinoma surgery pointed out that a low GNRI not only effectively predicts decreased postoperative overall survival and recurrence-free survival but is also closely related to an increased risk of early recurrence and extrahepatic metastasis (41).

Subgroup analysis suggested a potential interaction between sex and the association between GNRI and ICU admission risk. The higher baseline ICU admission rate observed in males (14.7% vs. 8.0%) suggests that, although the relative risk increase may be similar between sexes, the absolute risk burden could differ. One possible explanation for this observation is sex-related differences in body composition: myosteatosis has been reported to be associated with malnutrition only in females, while muscle loss appears more prominent in males (42). Additionally, sex hormones play a role in regulating inflammatory and immune responses, which may affect postoperative recovery. Estrogen has been suggested to exhibit anti-inflammatory properties, whereas testosterone may influence inflammatory pathways, with evidence indicating that older males may heal more slowly than females (43, 44). These hormonal factors might theoretically influence the threshold at which nutritional deficits lead to postoperative complications requiring intensive care. Previous studies have also explored whether the prognostic value of GNRI differs by sex. Liu et al. found that GNRI was a significant independent predictor of mortality in older trauma patients admitted to the ICU, with no significant sex predominance difference between the mortality and survival groups (45, 46). Shoji et al. reported that both GNRI and sex were significantly associated with postoperative complications and overall survival in elderly patients with non-small cell lung cancer (47). Taken together, although GNRI remains a valid predictor for both sexes, future research may explore whether sex-specific cutoff values or risk stratification strategies warrant consideration. Importantly, given that this interaction finding emerged from post-hoc subgroup analysis, it should be interpreted as hypothesis-generating and exploratory, requiring prospective validation.

This study applied GNRI to the predictive assessment of the specific perioperative event “ICU admission” in postoperative patients with gastrointestinal tumors, systematically validating the predictive value of GNRI for high-intensity healthcare resource utilization events. GNRI has the characteristics of being simple, objective, and easily obtainable, requiring no complex questionnaires, expensive equipment, or special anthropometric measurements, making it suitable for healthcare institutions at all levels. It can effectively identify high-risk patients who appear stable preoperatively but actually have insufficient physiological reserve and are prone to postoperative decompensation, thereby providing a basis for precise preoperative risk stratification, rational allocation of medical resources, and clinical treatment strategies.

This study has several limitations. First, although multiple adjustments were made, the retrospective observational design cannot completely rule out residual bias caused by unmeasured confounding factors. Second, although the INSPIRE database is substantial in scale, it lacks details such as types of postoperative complications, making it difficult to fully elucidate the specific pathophysiological pathways through which low GNRI leads to ICU admission. Third, the database lacks dynamic nutritional indicators (e.g., recent weight loss rate, dietary intake) and does not distinguish between planned and unplanned ICU admissions, as our definition captured all first-time postoperative ICU admissions. Fourth, we did not examine postoperative drug dosing or adverse drug events, so we cannot rule out the possibility that higher GNRI (and consequently higher albumin) might alter the distribution of highly protein-bound drugs, potentially requiring higher dosages and increasing adverse effect risk. Future prospective studies are needed to further reveal the causal relationships and specific mechanisms involved.

## 5 Conclusion

Preoperative low GNRI is an independent predictor of ICU admission risk in patients after gastrointestinal tumor surgery. A non-linear dose–response relationship exists between GNRI and ICU admission risk, with the risk significantly increasing when GNRI falls below 89. The severe nutritional risk category (GNRI < 82) provides a clinically meaningful threshold for identifying high-risk patients. As a comprehensive indicator integrating nutritional and inflammatory status, GNRI can provide evidence-based support for perioperative risk stratification and medical resource optimization in patients with gastrointestinal tumors.

## Data Availability

Publicly available datasets were analyzed in this study. This data can be found at: https://www.nature.com/articles/s41597-024-03517-4 INSPIRE (Informative Surgical Patient dataset for Innovative Research Environment).

## Supplementary material

SUPPLEMENTARY FIGURE S1 Pair wise correlation matrix of the included variables.

SUPPLEMENTARY FIGURE S2 Visualization of baseline characteristics stratified by GNRI group.

SUPPLEMENTARY FIGURE S3 Changes in standardized mean differences of variables before and after propensity score adjustment.
