# Supplementary figures and images for: Preoperative low Geriatric Nutritional Risk Index increases intensive care unit admission risk in patients undergoing gastrointestinal tumor surgery
Source: Front Nutr. 2026 May 28;13:1731167. doi: 10.3389/fnut.2026.1731167 (PMC13254269; doi:10.3389/fnut.2026.1731167)

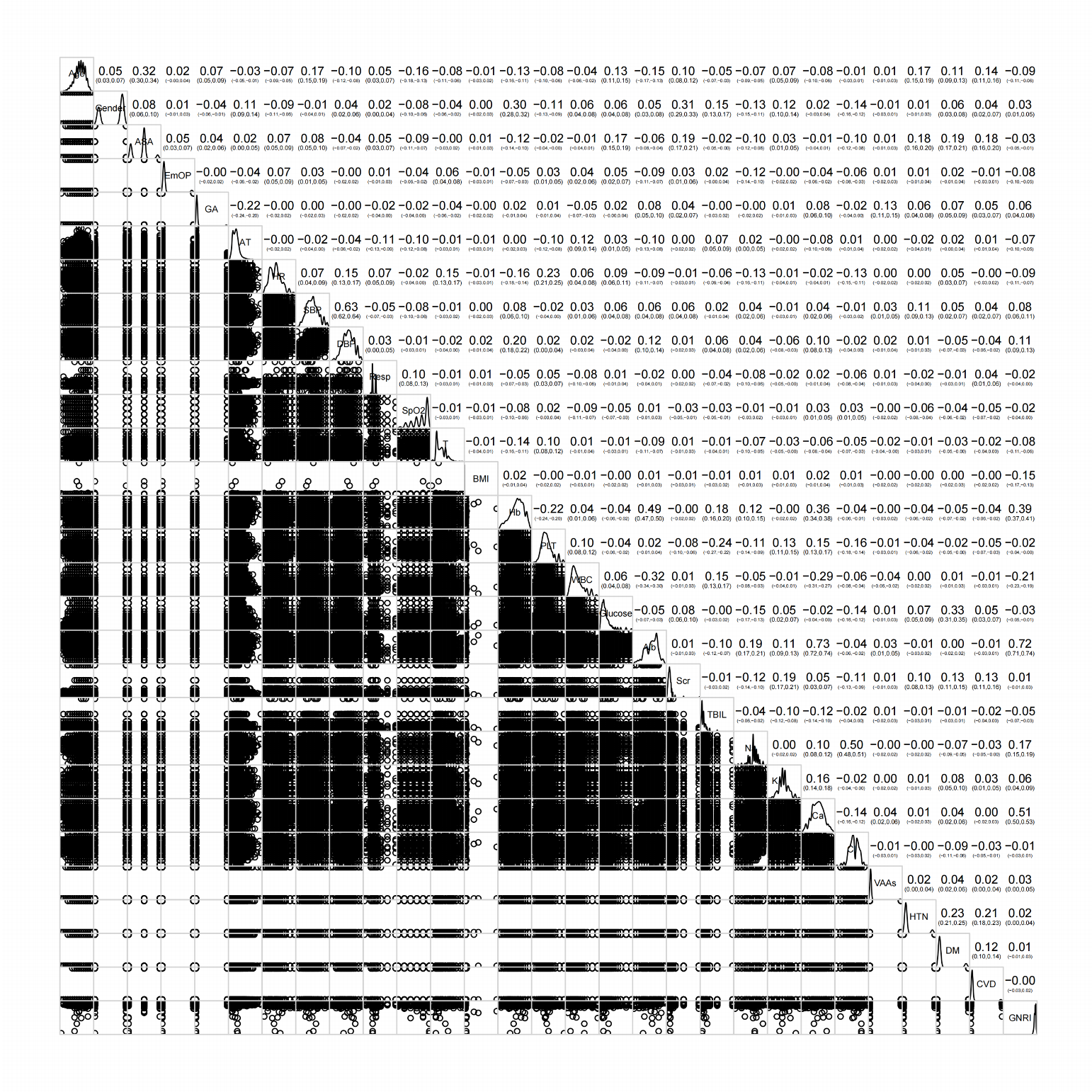

Supplement: SUPPLEMENTARY FIGURE S1 — Pair wise correlation matrix of the included variables. [file Image_1.tif]

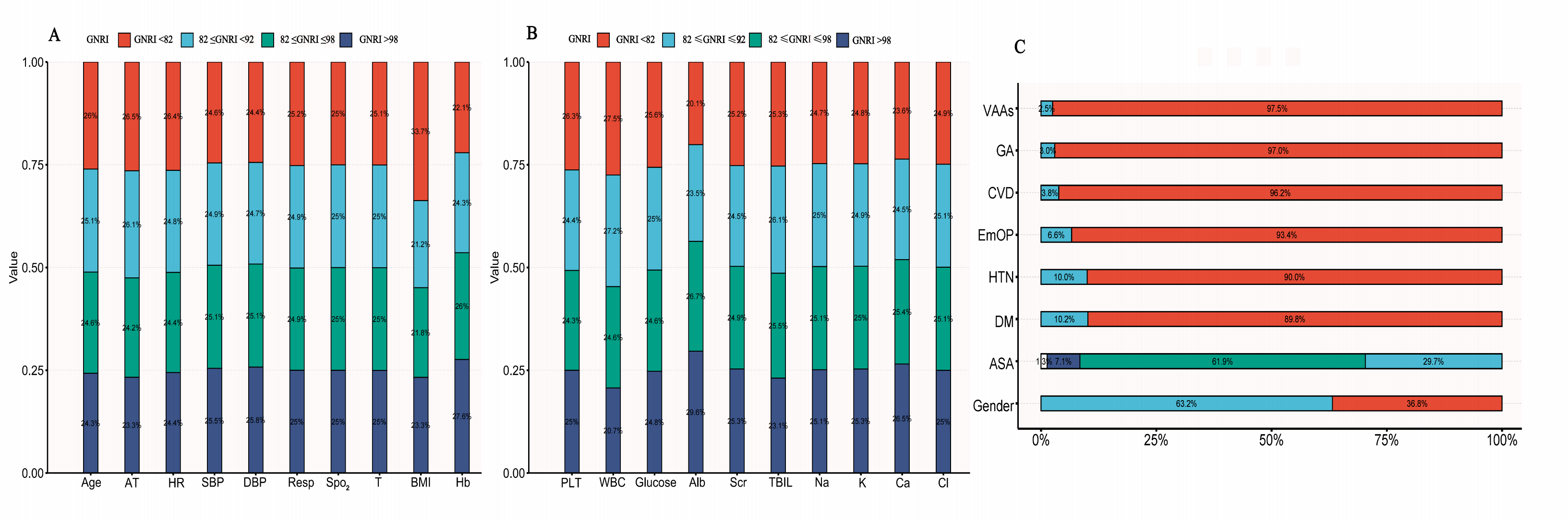

Supplement: SUPPLEMENTARY FIGURE S2 — Visualization of baseline characteristics stratified by GNRI group. [file Image_2.tif]

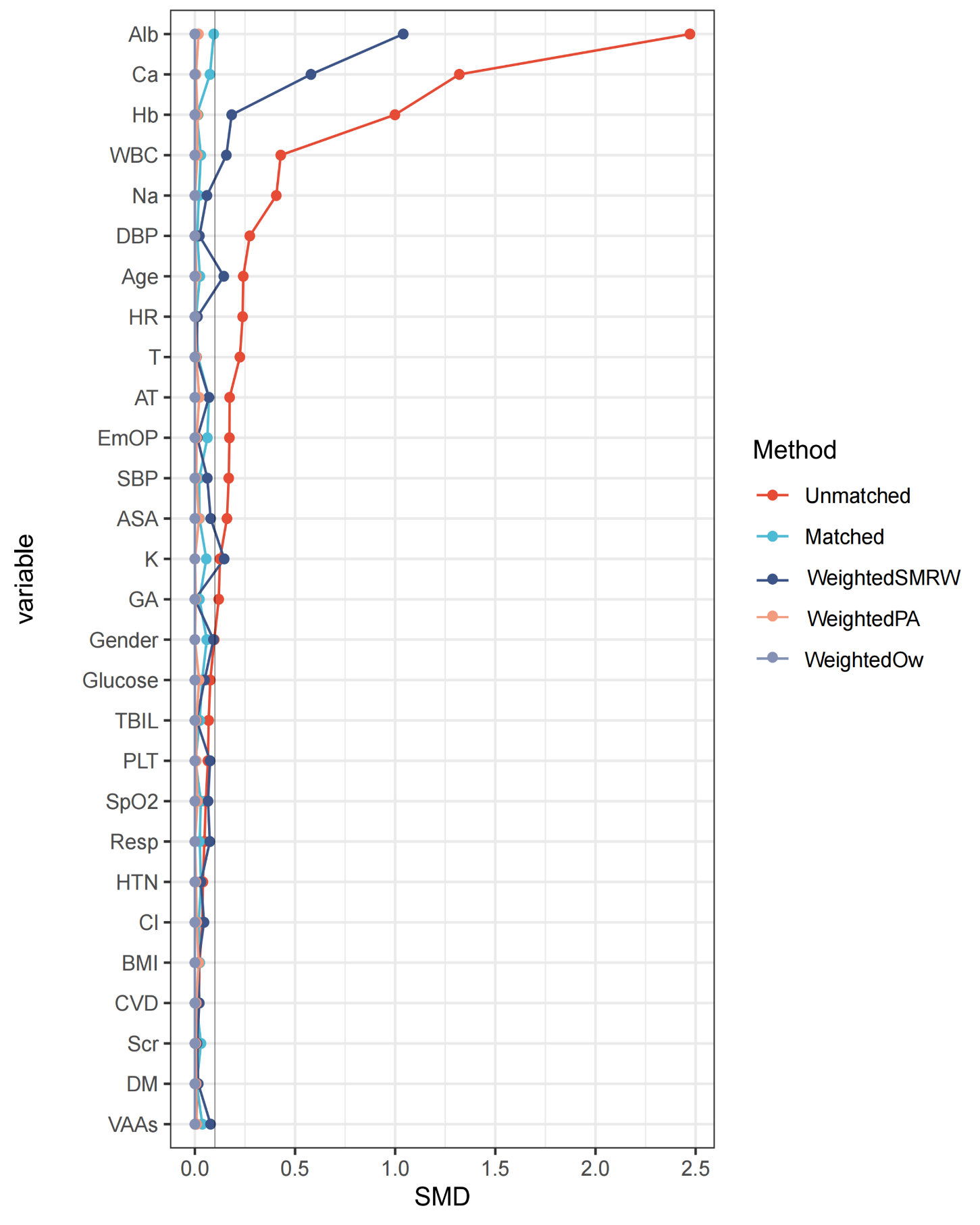

Supplement: SUPPLEMENTARY FIGURE S3 — Changes in standardized mean differences of variables before and after propensity score adjustment. [file Image_3.tif]
