# Supplementary material for: Preoperative low Geriatric Nutritional Risk Index increases intensive care unit admission risk in patients undergoing gastrointestinal tumor surgery
Source: Front Nutr. 2026 May 28;13:1731167. doi: 10.3389/fnut.2026.1731167 (PMC13254269; doi:10.3389/fnut.2026.1731167)
Supplement: Supplementary file 4 [file Table_1.docx]

**TABLE S1** Results of Covariate Selection

| **Term1** | **Change.percentage1** | **Change.percentage2** | **GVIF** | **DF** | **GVIF^(1/(2*Df))** | **colinearity** | **select** |
| --- | --- | --- | --- | --- | --- | --- | --- |
| **Crude/Full** | Ref. | Ref. | 2.302 | 1 | 1.517 | 0 | Ref. |
| **Age** | -25 | -7.1 | 1.286 | 1 | 1.134 | 0 | Yes |
| **Gender** | 5.8 | 3.1 | 1.224 | 1 | 1.107 | 0 | No |
| **ASA** | -23.1 | -5.2 | 1.212 | 2 | 1.049 | 0 | Yes |
| **EmOP** | -12.3 | 2.9 | 1.078 | 1 | 1.038 | 0 | Yes |
| **GA** | -7 | 0.4 | 1.022 | 1 | 1.011 | 0 | No |
| **HR** | -9.2 | 1.4 | 1.202 | 1 | 1.096 | 0 | No |
| **SBP** | -3.4 | 0.2 | 1.882 | 1 | 1.372 | 0 | No |
| **DBP** | -18.2 | 0.7 | 1.91 | 1 | 1.382 | 0 | Yes |
| **Resp** | -4.9 | 0 | 1.059 | 1 | 1.029 | 0 | No |
| **SpO_2_** | -1.8 | -3.3 | 1.064 | 1 | 1.031 | 0 | No |
| **T** | -6.9 | 2.1 | 1.078 | 1 | 1.038 | 0 | No |
| **Glucose** | 0.4 | -0.4 | 1.178 | 1 | 1.085 | 0 | No |
| **Scr** | -2.3 | -1.2 | 1.229 | 1 | 1.109 | 0 | No |
| **K** | 3.1 | 0.1 | 1.138 | 1 | 1.067 | 0 | No |
| **Na** | -29 | 4.4 | 1.588 | 1 | 1.26 | 0 | Yes |
| **Hb** | -34.2 | 1.9 | 1.84 | 1 | 1.357 | 0 | Yes |
| **WBC** | 8.5 | 0.4 | 1.292 | 1 | 1.137 | 0 | No |
| **PLT** | 0.1 | -0.2 | 1.344 | 1 | 1.159 | 0 | No |
| **Cl** | -4.1 | 0.6 | 1.503 | 1 | 1.226 | 0 | No |
| **Ca** | 26.2 | 1 | 2.313 | 1 | 1.521 | 0 | Yes |
| **Alb** | -39 | -72.1 | 3.996 | 1 | 1.999 | 0 | Yes |
| **TBIL** | 1.8 | 0.5 | 1.152 | 1 | 1.073 | 0 | No |
| **AT** | -14.3 | -17.7 | 1.156 | 1 | 1.075 | 0 | Yes |
| **BMI** | 29.8 | -47.7 | 1.108 | 1 | 1.052 | 0 | Yes |
| **VAAs** | 1.7 | -0.6 | 1.01 | 1 | 1.005 | 0 | No |
| **HTN** | 2.6 | 0.5 | 1.116 | 1 | 1.056 | 0 | No |
| **DM** | 2.9 | -0.3 | 1.207 | 1 | 1.099 | 0 | No |
| **CVD** | -0.7 | -0.5 | 1.098 | 1 | 1.048 | 0 | No |

**Abbreviations:** ASA classification, American Society of Anesthesiologists classification; EmOP, Emergency Operation; GA, General anesthesia; AT, Anesthesia Time; HR, Heart rate; SBP, systolic blood pressure; DBP, diastolic blood pressure; Resp, respiratory; SpO_2_, pulse oximetry derived oxygen saturation; T, Temperature; BMI, body mass index; Hb, hemoglobin; PLT, Platelet Count; WBC, white blood cell; Alb, albumin; Scr, serum creatinine; TBIL, total bilirubin; Na, Sodium; K, Potassium; Ca, Calcium; Cl, Chlorine; VAAs, Vasoactive Agents; HTN, Hypertension; DM, diabetes mellitus; CVD, Cardiovascular Disease.
