# Supplementary material for: Preoperative low Geriatric Nutritional Risk Index increases intensive care unit admission risk in patients undergoing gastrointestinal tumor surgery
Source: Front Nutr. 2026 May 28;13:1731167. doi: 10.3389/fnut.2026.1731167 (PMC13254269; doi:10.3389/fnut.2026.1731167)
Supplement: Supplementary file 5 [file Table_2.DOCX]

**TABLE S2** | **Univariate analysis of ICU admission in patients undergoing gastrointestinal tumor surgery.**

| **Variables** | **OR(95%CI)** | ***P-*value** |
| --- | --- | --- |
| **Age** | 1.06 (1.05~1.07) | <0.001 |
| **Gender** | 1.99 (1.72~2.3) | <0.001 |
| ****ASA classification**** |  |  |
| Ⅰ | Ref |  |
| Ⅱ | 2.59 (2.14~3.13) | <0.001 |
| ≥Ⅲ | 13.24 (10.48~16.72) | <0.001 |
| ****EmOP**** | 2.28 (1.86~2.79) | <0.001 |
| ****GA**** | 0.05 (0.01~0.21) | <0.001 |
| **AT** | 11.95 (10.03~14.23) | <0.001 |
| **HR** | 1.42 (1~2.02) | 0.051 |
| **SBP (mmHg)** | 1 (0.63~1.59) | 0.988 |
| **DBP (mmHg)** | 0.19 (0.13~0.3) | <0.001 |
| **Resp (bpm)** | 1.01 (0.95~1.06) | 0.766 |
| **SpO_2_** | 0.85 (0.82~0.88) | <0.001 |
| **T** | 1.14 (0.97~1.35) | 0.105 |
| **BMI** | 0.95 (0.93~0.96) | <0.001 |
| **Hb** | 0.9 (0.87~0.93) | <0.001 |
| **PLT** | 0.58 (0.48~0.7) | <0.001 |
| **WBC** | 0.99 (0.97~1.01) | 0.258 |
| **Glucose** | 2.86 (2.31~3.54) | <0.001 |
| **Alb** | 0.72 (0.64~0.81) | <0.001 |
| **Scr** | 1.7 (1.53~1.9) | <0.001 |
| **TBIL** | 0.89 (0.77~1.02) | 0.084 |
| **Na** | 0.91 (0.89~0.93) | <0.001 |
| **K** | 1.52 (1.3~1.78) | <0.001 |
| **Ca** | 0.97 (0.86~1.08) | 0.549 |
| **Cl** | 0.97 (0.95~0.99) | <0.001 |
| **VAAs** | 1.65 (1.17~2.32) | 0.004 |
| **HTN** | 1.6 (1.33~1.92) | <0.001 |
| **DM** | 2.22 (1.87~2.64) | <0.001 |
| **CVD** | 3.61 (2.86~4.57) | <0.001 |

**Abbreviations:** ASA classification, American Society of Anesthesiologists classification; EmOP, Emergency Operation; GA, General anesthesia; AT, Anesthesia Time; HR, Heart rate; SBP, systolic blood pressure; DBP, diastolic blood pressure; Resp, respiratory; SpO_2_, pulse oximetry derived oxygen saturation; T, Temperature; BMI, body mass index; Hb, hemoglobin; PLT, Platelet Count; WBC, white blood cell; Alb, albumin; Scr, serum creatinine; TBIL, total bilirubin; Na, Sodium; K, Potassium; Ca, Calcium; Cl, Chlorine; VAAs, Vasoactive Agents; HTN, Hypertension; DM, diabetes mellitus; CVD, Cardiovascular Disease.
