# Supplementary material for: Preoperative low Geriatric Nutritional Risk Index increases intensive care unit admission risk in patients undergoing gastrointestinal tumor surgery
Source: Front Nutr. 2026 May 28;13:1731167. doi: 10.3389/fnut.2026.1731167 (PMC13254269; doi:10.3389/fnut.2026.1731167)
Supplement: Supplementary file 6 [file Table_3.DOCX]

**TABLE S3** | **Sensitivity analysis of the association between GNRI and ICU admission based on multiple imputation.**

| **Variable** | **Crude model** | | **Model Ⅰ** | | **Model Ⅱ** | | **Model Ⅲ** | |
| --- | --- | --- | --- | --- | --- | --- | --- | --- |
|  | **OR (95% CI)** | ***P-*value** | **OR (95% CI)** | ***P-*value** | **OR (95% CI)** | ***P-*value** | **OR (95% CI)** | ***P-*value** |
| **GNRI (continuous)** | 1.01 (1.01~1.02) | <0.001 | 1.01 (1.01~1.02) | <0.001 | 1.02 (1.01~1.03) | <0.001 | 1.02 (1.01~1.03) | <0.001 |
| GNRI quartiles |  |  |  |  |  |  |  |  |
| **GNRI >98** | **Ref** |  | **Ref** |  | **Ref** |  | **Ref** |  |
| **92 ≤GNR ≤98** | 0.98 (0.82~1.16) | 0.798 | 0.94 (0.79~1.13) | 0.519 | 1.05 (0.81~1.37) | 0.719 | 1.05 (0.81~1.37) | 0.695 |
| **82 ≤GNRI <92** | 1.04 (0.88~1.22) | 0.643 | 0.94 (0.79~1.11) | 0.457 | 0.92 (0.63~1.35) | 0.675 | 0.93 (0.63~1.36) | 0.699 |
| **GNRI <82** | 2.32 (1.92~2.79) | <0.001 | 1.89  (1.55~2.3) | <0.001 | 1.81 (1~3.28) | 0.05 | 1.82 (1.01~3.29) | 0.048 |
| **P for trend** |  | <0.001 |  | <0.001 |  | 0.678 |  | 0.649 |

Notes: Crude model was not adjusted.

Model 1 was adjusted for age + Gender.

Model 2 was adjusted for model 1 + **ASA classification** + **EmOP** + **GA** + AT + HR + PLT + DBP + SpO_2_ + Hb + BMI + Glucose + Alb + Scr + TBIL + Na + K + Ca + Cl + VAAs + HTN + DM + CVD.

Model 3 was adjusted for model 2 + SBP + Resp + T + WBC.

**Abbreviations:** ASA classification, American Society of Anesthesiologists classification; EmOP, Emergency Operation; GA, General anesthesia; AT, Anesthesia Time; HR, Heart rate; SBP, systolic blood pressure; DBP, diastolic blood pressure; Resp, respiratory; SpO_2_, pulse oximetry derived oxygen saturation; T, Temperature; BMI, body mass index; Hb, hemoglobin; PLT, Platelet Count; WBC, white blood cell; Alb, albumin; Scr, serum creatinine; TBIL, total bilirubin; Na, Sodium; K, Potassium; Ca, Calcium; Cl, Chlorine; VAAs, Vasoactive Agents; HTN, Hypertension; DM, diabetes mellitus; CVD, Cardiovascular Disease.
